# Parenting Stress and Couple Relationship Quality Among Transgender and Nonbinary Parents: The Roles of Discrepancy in Division of Childcare Labor and Gender Identity

**DOI:** 10.1111/famp.70141

**Published:** 2026-03-26

**Authors:** Shixin Fang, Samantha L. Tornello, Emma Spadaro

**Affiliations:** ^1^ Department of Human Development and Family Studies The Pennsylvania State University University Park Pennsylvania USA

**Keywords:** division of childcare labor, parenting stress, relationship quality, transgender and nonbinary parents

## Abstract

A substantial population of transgender and nonbinary (TNB) individuals is becoming parents. However, research on how TNB parents navigate parenthood, family dynamics, and couple functioning remains limited. This study investigated the association between parenting stress and relationship quality and tested the mediating role of discrepancy in the division of childcare labor (i.e., satisfaction with the current allocation of childrearing tasks) and the moderating role of gender identity (i.e., transgender man and woman vs. nonbinary) in an international sample of 228 TNB parents primarily from the United States. Results of structural equation modeling identified a conditional indirect pathway: Higher levels of parenting stress were negatively associated with lower couple relationship quality through greater discrepancy in the division of childcare labor only among nonbinary parents rather than transgender man and woman parents. The findings of this study shed light on the understudied intricacies of TNB parents' intersectional experiences of their gender identity, intimate relationships, and parenthood. The practical significance of this study lies in its potential to inform targeted intervention programs that ultimately enhance couple relationship well‐being through the development of a more desirable division of childcare labor considering the nuanced experiences of TNB parents.

## 1 Introduction

Transgender and nonbinary (TNB) people are broadly defined as individuals whose gender identity does not conform to the cultural and social expectations of the sex they were assigned at birth (American Psychological Association 2015). With clinical de‐pathologization and sociocultural shifts, TNB individuals experience greater societal visibility than in previous decades (Tebbe and Budge 2022). Still, TNB people experience gender‐related discrimination and stigma (Hendricks and Testa 2012), which are associated with disproportionately high risks of health disparities (Brown and Jones 2016; Connolly and Gilchrist 2020). According to the online EU‐LGBTI II Survey conducted across 28 European countries (European Union Agency for Fundamental Rights 2021), an average of 19% of TNB individuals are actively involved in raising a child with a romantic partner. In the United States, approximately 0.5%–2% of adults self‐identify as TNB in the USA (Flores et al. 2016; Herman et al. 2022), and an estimated 15% to 50% of TNB individuals are parents (Carone et al. 2021; James et al. 2016; Stotzer et al., 2014; Walls et al. 2019). Despite the considerable population of TNB households with children, few studies have explored their experiences as parents, family dynamics, and couple functioning.

The existing literature has demonstrated that couple relationship quality tends to decline over the transition to parenthood, regardless of couples' gender composition (Belsky and Rovine 1990; Goldberg et al. 2010; Goldberg and Sayer 2006). Among the potential precursors to detrimental changes in relationships, parenting stress is one of the most salient after couples become parents (Bradbury et al. 2000; Lavee et al. 1996). According to Abidin (1990), parents tend to experience parenting stress when confronted with a gap between the daily demands of parenting and the available resources that parents have to effectively address those demands. As couples are often dealing with childrearing tasks together, a sense of imbalance in responsibilities is likely to spill over into their relationship dynamics (Durtschi et al. 2017; Lavee et al. 1996). Mounting studies revealed an overall negative linkage between parenting stress and couple relationship well‐being among both heterosexual (Berryhill et al. 2016; Camisasca et al. 2014; Dong et al. 2022) and sexual minority couples (Horne et al. 2022). However, empirical work on the association between parenting stress and relationship quality is still limited in several ways.

Extant studies have predominantly centered on the generic and direct links between parenting stress and relational well‐being, with only a few seeking to identify the underlying mechanisms. For example, two studies based on samples of Chinese parenting couples both found that higher parenting stress contributed to lower marital quality through the emergence of parental depression (Ding et al. 2022; Dong et al. 2022). Prior research has identified an indirect pathway whereby parenting stress contributes to marital conflict through coparenting dynamics (Han and Lee 2020). The ecological model of coparenting posits that coparenting dynamics encompass four interrelated components: agreement or disagreement on childrearing, division of childcare labor, coparenting support or undermining, and the joint management of family interactions. More importantly, these coparenting features play an intermediate role within family systems and are closely tied to the adjustment of both parenting dimensions and couple dynamics (Feinberg 2003). It is plausible that one of coparenting dimensions, division of labor, serves as a mechanism linking parenting stress and relational well‐being. Specifically, the discrepancies in actual and ideal division of childcare responsibilities are a strong predictor of poorer couple relationship well‐being, and matters even more than the actual contribution by each partner (Tornello, Kruczkowski, and Patterson 2015; Tornello, Sonnenberg, and Patterson 2015).

Moreover, coparenting dynamics are studied predominantly among cisgender heterosexual couples. In these couples, coparenting is conventionally gendered, with mothers often taking on more childcare responsibilities than fathers (Bianchi et al. 2000; Coltrane 2000). While demands for fathers' active involvement have increased and more progressive gender‐role beliefs are associated with more positive coparenting relationships (Campbell 2023), a gendered division of labor where women remain responsible for the majority of household and childcare tasks still persists (Lachance‐Grzela and Bouchard 2010; Yavorsky et al. 2015). In contrast, TNB parents tend to divide childcare labor in a more egalitarian fashion than their cisgender counterparts (Tornello 2020), although family dynamics differ across gender identities within TNB parent groups (Riskind and Tornello 2022). Specifically, nonbinary adults are less likely than binary transgender individuals (i.e., those who primarily identify as transgender men or women) to conform to hegemonic gender norms (Bradford and Catalpa 2019; Catalpa et al. 2019). This variation in gender conformity relates to household power dynamics: for instance, parents who present with more masculine appearances or demeanors tend to perform less unpaid caregiving labor (Pollitt et al. 2018). Together, these distinctions suggest that the pathway between parenting stress and relationship quality via division of childcare labor discrepancy varies across different gender groups within TNB parents.

Taken together, this study sought to investigate the association between parenting stress and couple relationship quality among TNB parents, and to test the mediating role of discrepancy in the division of childcare labor and the moderating role of gender identity (i.e., transgender man and woman vs. nonbinary) in such association.

### 1.1 Theoretical Framework

The theoretical foundation for this study was based on an integration of the ecological model of coparenting (Feinberg 2003), the Vulnerability‐Stress‐Adaptation (VSA) model (Karney and Bradbury 1995), and the conceptual model of decentering heteronormativity (Oswald et al. 2005). First, the ecological model of coparenting posits that coparenting dynamics serve as a central mechanism through which individual, family, and extrafamilial risk factors influence family outcomes, including mediating the linkage between parental adjustment and interparental relationships (Feinberg 2003). Within this framework, the features captured by parenting stress, such as distress related to the strain of parenthood (Abidin 1990), may be associated with specific aspects of coparenting, including parents' perceived discrepancies in the division of childrearing tasks, which in turn may contribute to overall relationship well‐being.

With the VSA model (Karney and Bradbury 1995) as a lens, stressful events that partners encounter are conceptualized as shaping their behavioral exchanges with each other, which in turn contribute to their perceptions of relationship quality. The VSA model suggests an essential indirect pathway for relationship distress: external stress arising from outside the romantic relationship (extra‐dyadic stress) may trigger tensions within the relationship (intra‐dyadic stress), decreasing interaction quality and ultimately leading to overall negative relational outcomes (Randall and Bodenmann 2009; Randall et al. 2023). As such, it seems warranted to expect that exposure to parenting stress, as an extra‐dyad stressor, may negatively impact TNB parents' relationship quality through giving rise to destructive within‐dyad dynamics, such as discrepancies in the division of childcare labor.

Oswald et al. (2005) proposed a model of decentering heteronormativity for family scholarship to disentangle the socially constructed facets of gender, sexuality, and family binaries that contribute to heteronormativity. To challenge deep‐rooted gender conventionality, researchers were encouraged to move beyond assumptions of gender essentialism and focus on more complex gender experiences. Consistent with the idea of “complex gendering” (Oswald et al. 2005), we centered queer and gender‐diverse people's experiences by investigating how multiple family subsystems among TNB parents subvert and resist stereotypes related to sex assigned at birth and parenting roles and captured the varied coparenting dynamics between gender binary and nonbinary groups within TNB parents. With the acknowledgment that the categorization of gender as binary or nonbinary may overgeneralize the diverse spectrum within the TNB group, our examinations serve as the initial exploratory step to better understand the nuanced intersection of gender and parenthood.

### 1.2 Parenting Stress and Couple Relationship Quality

According to Abidin (1990), parenting stress is a multidimensional situational demand consisting of the sense of restriction and deprivation associated with parenthood, parents' perceptions of children's temperament or demanding behaviors, and parents' perceived quality of interactions with their children. When there is an imbalance between the demands of parenting and the resources available to parents, parents tend to encounter parenting stress (Cooper et al. 2009). Previous evidence showed that parenting stress was a robust predictor of negative couple interactions (Berryhill et al. 2016; Camisasca et al. 2014; Chester and Blandon 2016; Durtschi et al. 2017; Hartley et al. 2018; Lavee et al. 1996). For example, using 3‐year longitudinal dyadic data drawn from 848 ethnically diverse heterosexual couples in their transition to parenthood, Durtschi et al. (2017) observed an actor effect that fathers' early parenting stress later predicted lower relationship quality reported by themselves.

### 1.3 Discrepancy in the Division of Childcare Labor as a Potential Mediator

Discrepancy in the division of childcare labor may serve as the mediating role in the association between parenting stress and couple relationship quality. The discrepancy in the division of childcare labor reflected the difference between currently shared participation in childcare tasks and desired shared participation (Cowan and Cowan 1988), which is a key aspect of coparenting dynamics (Feinberg 2003). The indirect effect of parenting stress on relationship quality among TNB parents via discrepancies in the division of childcare labor is a deductive proposition derived from the VSA model, which posits that external stressors originating outside the couple relationship spill over into the relationship through maladaptive dyadic processes such as mutual alienation (Karney and Bradbury 1995; Randall et al. 2023).

Empirical evidence also supports this postulation. Much prior work linking parenting stress to the division of childcare labor has focused on broader coparenting dynamics, of which division of labor is a critical indicator. For example, a cross‐lagged study has shown that parenting stress reliably predicted lower coparenting quality observed across three, six, and twelve months postpartum (Kang et al. 2022). Likewise, in a study of 1100 heterosexual parents from the Netherlands, women's dissatisfaction with the current division of childcare labor increased when parenting demands rose (Koster et al. 2022).

In terms of the association between coparenting and relationship well‐being, researchers identified a reciprocal link (Le et al. 2016; Schoppe‐Sullivan et al. 2004). However, Schoppe‐Sullivan et al. (2004) found that early supportive or undermining coparenting behaviors were consistently indicative of later marital interactions, but not vice versa. Extending this work to TNB parents, greater discrepancies between the current and ideal division of childcare labor, rather than the current division of childcare labor, were predictive of poorer relationship quality (Tornello 2020). Hence, it is conceivable that parenting stress may contribute to greater discrepancies in division of childcare labor, which in turn may negatively impact couple relationship quality.

### 1.4 Gender Identity as a Potential Moderator

An expansive range of gender identities, expressions, and experiences exists among TNB people. Despite this heterogeneity, prior work has primarily framed transgender identity within a binary, focusing on transgender men and transgender women, in which researchers have identified meaningful group differences in mental health, substance use, and healthcare experiences (James et al. 2016; Lefevor et al. 2019). More recently, distinctions between binary and nonbinary identities within TNB communities have garnered burgeoning empirical attention, especially when examining the extent to which TNB individuals endorse dominant gender ideologies and how they navigate prevailing gender norms (Bradford and Catalpa 2019; Catalpa et al. 2019). Thus, differences in parenting experiences between binary and nonbinary parents are particularly relevant to the highly gendered practice of dividing childcare labor, such that the adverse implications of parenting stress on discrepancy in the division of childcare labor might vary from binary to nonbinary parents. Specifically, gender binary parents referred to gender‐diverse parents whose current gender identity differed from their assigned sex at birth and identified primarily as woman or man, while nonbinary parents referred to parents whose current gender identity did not align with their assigned sex at birth and who identified outside or between a woman–man binary framework, including nonbinary, agender, genderqueer, gender nonconforming, and gender fluid (American Psychological Association 2015). Gender identity of nonbinary people may be neither feminine nor masculine, both feminine and masculine, or either feminine or masculine at separate times (Lefevor et al. 2019).

Emerging research highlights the nuances of gender ideology and practices between binary and nonbinary individuals within TNB populations (Bradford and Catalpa 2019; Catalpa et al. 2019). According to the measurement development studies of gender ideology among TNB people (Catalpa et al. 2019; McDermott et al. 2021), nonbinary individuals are inclined to hold less patriarchal beliefs about men and women, less endorsement for femininity norms, and more fluidity in gender expression than binary peers. Extending this to family processes, nonbinary parents may be less likely to have a preexisting pattern of domestic labor expectations based on gender roles (Riskind and Tornello 2022) and may advocate more for a fairer tenet in the division of labor than binary parents (Tornello 2020). When confronted with heightened parenting stress, nonbinary parents may encounter greater difficulty in coordinating a division of labor that aligns with their satisfaction compared to binary parents.

### 1.5 This Study

**FIGURE 1:**
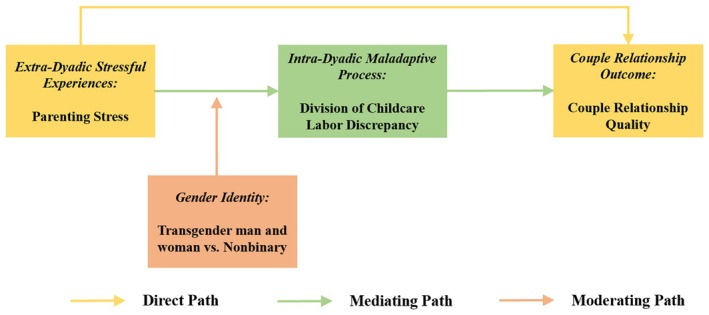
The conceptual model.

This study used cross‐sectional, self‐report survey data from 228 transgender and nonbinary parents residing in Westernized countries who were in romantic relationships. We sought to examine the association between extra‐dyadic stressful experiences (i.e., parenting stress) and couple relationship quality. We tested an intra‐dyadic maladaptive process of discrepancy in the division of childcare labor as a potential mediator that may account for this link. We also investigated whether the association between parenting stress and discrepancy in the division of childcare labor was moderated by TNB parents' gender identity. Ultimately, we expect to identify a conditional indirect pathway, such that TNB parents' experiences of parenting stress would be negatively associated with their relationship quality through a positive association with their perceived discrepancy in the division of childcare labor, and the indirect effect observed among nonbinary parents would be larger than that among transgender man and woman parents (see Figure 1 for a conceptual illustration). In sum, we had three hypotheses in our study:

*Greater parenting stress is associated with lower levels of couple relationship quality among TNB parents*.

*Greater parenting stress is indirectly associated with lower levels of couple relationship quality via greater discrepancy in the division of childcare labor*.

*Gender identity moderates the association between parenting stress and discrepancy in the division of childcare labor, such that the link is stronger among nonbinary than transgender man and woman groups*.

## 2 Method

### 2.1 Participants and Procedures

Participants were recruited for a large longitudinal study of transgender and nonbinary (TNB) parents through advertisements on social media and websites specifically targeting TNB parents and their families. The inclusion criteria for the study required that individuals identify with a gender identity that did not align with the cultural expectations of their assigned sex at birth, be at least 18 years old, and have at least one child. Interested individuals who met the inclusion criteria were required to either contact the PI (the second author) directly or complete an online onboarding form to provide an email address in order to receive a personalized study link and password (see Tornello et al. 2019, for recruitment details). The study was approved by the Institutional Review Board of The Pennsylvania State University (IRB# STUDY00005115). A total of 497 TNB parents completed the original survey. To further ensure data integrity, we systematically evaluated four indicators of potential fraudulent responding: (a) duplicate email addresses, (b) unusually short survey completion times, (c) answer patterns consistent with straightlining, and (d) the coherence and meaningfulness of required open‐text responses, such as consistency across occupation, weekly working hours, and income. No cases in the total sample raised concerns across these criteria. All responses were unique, completed within reasonable time frames, demonstrated variability in item responses, and included coherent open‐text information.

For the designated aims of this study, participants were excluded if they were not in a romantic relationship (*n* = 73), did not indicate their relationship status (*n* = 7), or their oldest child was older than 12 years of age (*n* = 152), as the parenting stress measure used was designed for parents of children aged 12 or younger (Abidin 1990). To ensure the independence of participants within couples, if both partners completed the survey, only one individual per couple was retained (*n* = 37), with the second respondent (chronologically) excluded. This selection process resulted in a final sample of 228 TNB parents.

The final sample had an average age of 35.30 years (SD = 6.67) and was predominantly White/Caucasian American (89.91%). Participants resided in multiple countries, with the majority living in the United States (73.68%) and smaller proportions residing in Canada (9.65%), the United Kingdom (6.14%), Australia (5.26%), and several other Westernized countries (5.26%; e.g., Germany, Finland, and Sweden). Almost half (*n* = 113, 49.56%) had only one child. The *mean* age of the participants' eldest child in the household was 5.32 years old (SD = 3.68). Many participants identified their gender as transgender women (20.18%) and transgender men (21.05%). Other gender identities extending beyond a binary framework included genderqueer (13.60%), nonbinary (11.40%), gender nonconforming (5.26%), gender fluid (3.51%), etc. Most participants identified as members of a sexual minority group (92.54%). Detailed demographic information for the final sample appears in Table S1–S2.

### 2.2 Measures

#### 2.2.1 Gender Identity

Participants were asked, “What is your current gender identity?” The following response options were provided: woman, man, transgender woman, transgender man, genderqueer, gender nonconforming, gender fluid, nonbinary, agender, bigender, choose not to label, and a self‐describe option with an open‐ended prompt. Participants were instructed to select a single response option. For those participants who identified within the experiences of binary genders (i.e., woman, man, transgender woman, and transgender man), we recoded their gender identity into a single category labeled *transgender man and woman*. Although the terms *transgender man and woman* and *binary transgender* refer to conceptually similar groups, and our rationale for examining gender differences draws on distinctions between binary and nonbinary identities documented in the TNB literature, we chose to use the terminology *transgender man and woman* when describing and discussing results in this study. This approach more accurately reflects participants' self‐selected identities and avoids imposing externally derived labels. Participants whose gender identities did not fall into binary gender terms (e.g., genderqueer, gender nonconforming, gender fluid, and nonbinary) were categorized into the *nonbinary* group.

For participants who provided a self‐described gender identity in an open‐text field (*n* = 14), we reviewed their written responses and recoded them into either the *transgender men and women* group or the *nonbinary* group. The open‐text responses included single‐word identities not listed among the response options (e.g., androgyne) as well as descriptions consisting of multiple labels. Participants whose open‐text descriptions included any identity within the nonbinary spectrum were coded as *nonbinary*; those whose descriptions consisted exclusively of binary‐identified terms (e.g., FTM, male, and transwoman) were coded as *transgender man and woman*. Participants who selected “choose not to label” (*n* = 1) were coded as missing for the recoded gender identity variable. Following this recoding procedure, the final sample included 122 transgender men and women parents and 105 nonbinary parents.

#### 2.2.2 Parenting Stress

The Parenting Stress Index‐Short Form (PSI‐SF; Abidin 1990) was used to measure the level and source of stress related to the responsibilities of being a parent. The PSI‐SF consists of 36 items along with measuring three factors: parental distress (PD), parent–child dysfunctional interaction (PCDI), and difficult child (DC), with each factor indicated by 12 items. The PD subscale centered on parents' sense of incompetence in childrearing (i.e., I often have the feeling that I cannot handle things well) and stress arising from parental role constraints (i.e., I don't enjoy things as I used to). The PCDI subscale quantified the emotional quality of parents' interactions with their children (i.e., Most times I feel that my child does not like me and does not want to be close to me). The DC subscale assessed the parents' perception of children's difficult temperament or non‐collaborative behaviors (i.e., My child's sleeping or eating schedule was much harder to establish than I expected). Thirty‐three out of the thirty‐six items asked participants to rate on a five‐point Likert scale from 1 = *strongly agree* to 5 = *strongly disagree*. Three items were on a five‐point Likert scale but used different anchors such as 1 = *Much harder than I expected* to 5 = *Much easier than I expected*, to respond to the statement such as: “I have found that getting my child to do something or stop doing something is:” Although the Parenting Stress Index has a three‐factor structure, it is common practice to sum all item scores to produce a total parenting‐stress score rather than a latent score (Ding et al. 2022; Tornello et al. 2011), and percentiles of this total score are often used as referral criteria for mental‐health services (Abidin 1990). Thus, parenting stress was represented by the sum score of all 36 items. Higher scores reflected greater parenting stress. Cronbach's α for the overall scale was 0.91.

#### 2.2.3 Discrepancy in the Division of Childcare Labor

The childcare subscale of Who Does What? (Cowan and Cowan 1990) was used to assess the actual and ideal division of labor in a household. Each item was scored on a nine‐point Likert scale in which 1 = *partner does it all*, 9 = *I do it all*, and 5 = *we both do this equally*. Participants were asked to rate two perceptions: how their current unpaid childcare division of labor (i.e., actual) is, and how they would like the unpaid childcare labor to be divided (i.e., ideal) for their first child. Based on the child's age, five different age‐adapted versions of childcare labor scales were utilized. The age ranges and example items were as follows: “feeding the baby” (0–6 months; 5.61% of participants), “choosing toys for children” (6–18 months; 11.21%), “arranging babysitters or childcare” (18–36 months; 10.28%), “reading to our children” (3–5 years; 20.09%), and “getting our child to and from school” (5–12 years; 48.13%). The number of items varied from 12 to 20 based on the age of the child. Cronbach's αs for different versions of actual and ideal childcare division of labor ranged from 0.78 to 0.96.

The actual and ideal childcare division of labor was represented by the mean score of all corresponding items. The discrepancy in the division of childcare labor was measured by the absolute difference score between the actual and ideal division of childcare labor. A score of zero indicated perfect satisfaction with the current division of childcare labor, while higher scores reflected a greater discrepancy between the actual and ideal division of labor.

#### 2.2.4 Couple Relationship Quality

Participants' relationship quality with their current romantic partner was measured by the 32‐item Dyadic Adjustment Scale (DAS; Spanier 1976). The DAS scale was comprised of four interrelated factors: dyadic consensus (i.e., career decisions), dyadic satisfaction (i.e., Do you confide in your mate?), dyadic cohesion (i.e., Calmly discuss something), and affectional expression (i.e., Not showing love). Item response options varied, with some items having six‐point Likert scales on agreement or frequency (0 = *always disagree* and 5 = *always agree*; 0 = *never* and 5 = *all the time*), or a two‐point scale in which 0 = *yes* and 1 = *no*. Subscale scores were generated by summing all corresponding item scores, with a higher score reflecting greater relational performance on that indicator. Cronbach's αs were 0.84, 0.85, 0.75, and 0.70 for consensus, satisfaction, cohesion, and affectional expression, respectively.

#### 2.2.5 Covariates

Participants were asked to respond to a series of demographic questions about themselves, their partners, and their children. According to previous empirical studies on division of labor (Tornello, Kruczkowski, and Patterson 2015; Tornello, Sonnenberg, and Patterson 2015), the number of children, the eldest child's age, and paid working hours per week were included in the proposed model as covariates.

### 2.3 Analytic Strategies

The item‐level missing amount for the study variables was from 0.00% to 28.51%. The results of Little's test (Little 1988) indicated a mechanism of missing completely at random (MCAR, *χ* ^2^ = 155.874, df = 140, *p* = 0.17) among the used variables. The full information maximum likelihood (FIML) estimation was used to handle missing data (Acock 2005). Hypotheses were tested by structural equation modeling in Mplus 8.3 (Muthén and Muthén 1998–2019). Relationship quality was specified as a latent variable, indicated by the total scores of each subscale, with the first factor loading fixed at one. All remaining study variables were observed.

**FIGURE 2:**
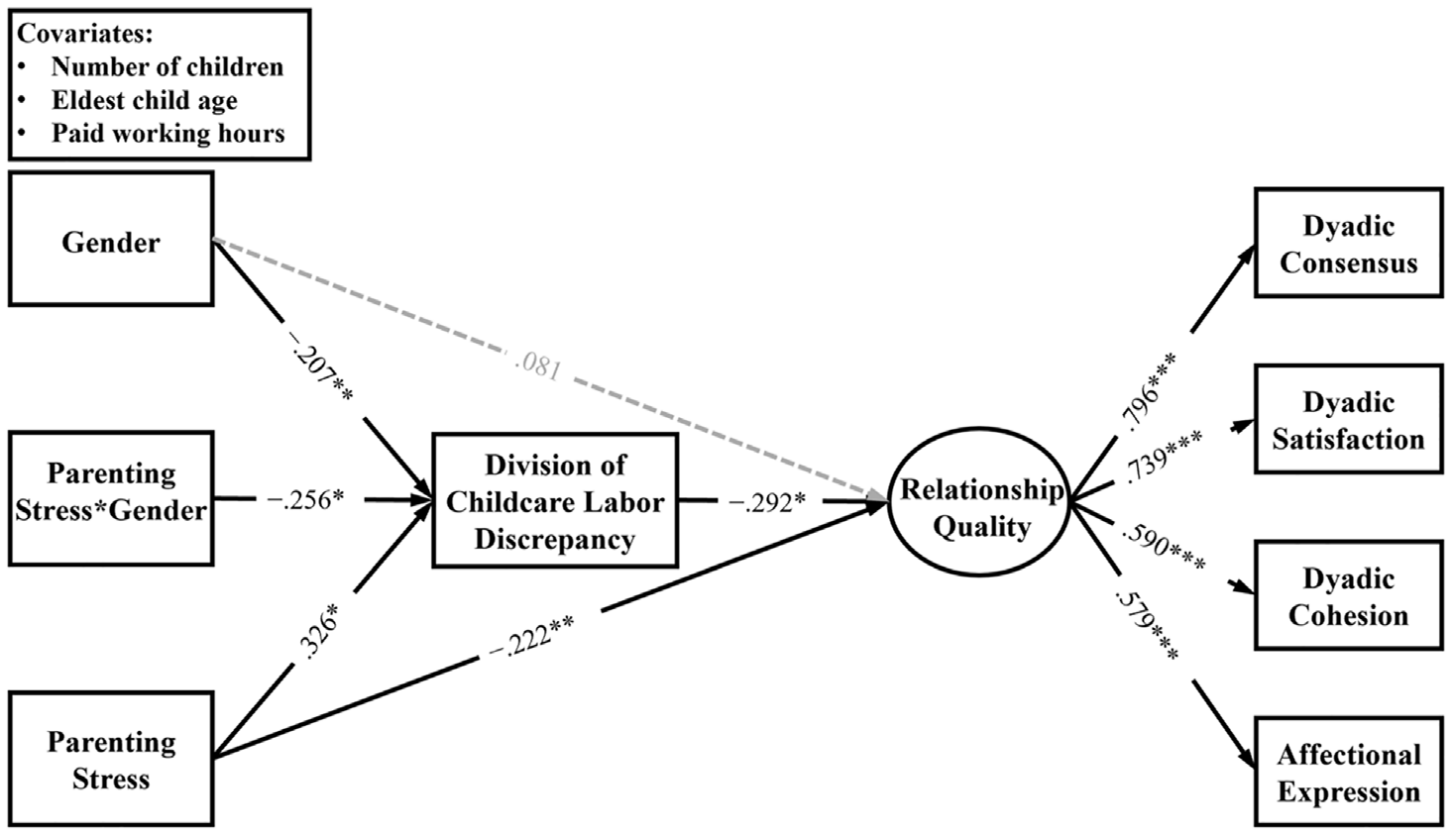
The moderated mediation model results. All estimated parameters were standardized with covariates loaded on both division of childcare labor discrepancy and relationship quality. To note, pathway lines with *p* > 0.05 were depicted in gray dash lines, whereas pathway lines with *p* < 0.05 are depicted in black solid lines. Gender identity was coded as 0 = transgender man and woman and 1 = nonbinary. *p < 0.05; **p < 0.01; ***p < 0.001 (two‐tailed).

As depicted in Figure 2, parenting stress and gender identity were included in the model to predict both the discrepancy in the division of childcare labor and relationship quality, with the discrepancy tested as a mediator in the association between parenting stress and relationship quality. In addition, a product term between parenting stress and gender identity was included to test the moderating role of gender identity in the links between parenting stress and discrepancy in the division of childcare labor. The product term of multiplying mean‐centered values of parenting stress and the original values of gender identity (0 = nonbinary, 1 = transgender man and woman) was created. To probe the identified interactive effect, simple slopes for nonbinary and transgender man and woman parents were estimated. We formally tested indirect (mediation) and conditional indirect (moderated‐mediation) effects using structural equation modeling combined with nonparametric bootstrapping. Conditional (group‐specific) indirect effects were computed as the product of the estimates of the path from predictor to mediator (a‐path) and the path from mediator to outcome (b‐path) for each level of the moderator. Because indirect‐effect sampling distributions are often nonnormal, we used bias‐corrected bootstrap confidence intervals (5000 resamples) to estimate the conditional indirect effects and their 95% CIs; an effect was considered statistically significant if the bootstrap CI did not include zero (Preacher et al. 2007). The magnitude of the effect size of the indirect effect was determined by Kenny's (2012) criteria. Both relationship quality and discrepancy in the division of childcare labor were regressed on covariates.

### 2.4 Sensitivity Analyses

Although our model was grounded in integrated theoretical propositions, we conducted two sets of sensitivity analyses to identify a final model that was both theoretically meaningful and statistically parsimonious. The first set of sensitivity analyses assessed whether combining transgender men and transgender women into a single category masked empirical differences between these groups. Using the subsample of transgender man and woman parents, we conducted linear regressions to test mean differences of study variables between transgender men (coded as 1; *n* = 58) and transgender women (coded as 0; *n* = 64). Regression results indicated no significant differences for parenting stress (*b* = 3.201, SE = 4.182, *p* = 0.446), discrepancy in division of childcare labor (*b* = 0.071, SE = 0.120, *p* = 0.553), or relationship quality (*b* = 3.067, SE = 3.179, *p* = 0.337). These tests suggested that the distinct family dynamics in relation to childcare division were unlikely to be driven by differences between transgender men and transgender women in our sample and were more likely to reflect differences between nonbinary parents and transgender man and woman parents.

The second set of sensitivity analyses examined whether gender identity moderated all key paths in the mediation model—specifically, the paths from parenting stress to division of childcare labor discrepancy, from division of childcare labor discrepancy to relationship quality, and the direct path from parenting stress to relationship quality. We performed a multigroup SEM analysis comparing nonbinary and transgender man and woman parents, starting with an unconstrained model and then imposing equality constraints on individual paths sequentially (see Table S2 for model comparison steps). *χ* ^2^ difference tests were used to determine whether constraining each path significantly worsened model fit. Results indicated that only constraining the path from parenting stress to division of childcare labor discrepancy significantly deteriorated model fit, suggesting that gender identity moderates only this association.

## 3 Results

Table 1 presents means, standard deviations, and zero‐order bivariate correlations among study variables. The structural equation model was used to examine the mediating role of discrepancy in the division of childcare labor in the link between parenting stress and relationship quality, and the moderating role of gender identity in the association between parenting stress and division of childcare discrepancy among TNB parents. Standardized coefficients for the structural equation model are displayed in Figure 2. The overall fit indices indicated that our hypothesized model fit the data well (Kline 2016): *χ*(24)^2^ = 30.071, *p* = 0.182, RMSEA [90% CI] = 0.033 [0.000, 0.067], CFI = 0.971, TLI = 0.951, SRMR = 0.036.

**TABLE 1:** Descriptive statistics and zero‐order bivariate correlations among study variables.

	Mean	SD	1	2	3	4	5	6	7
*Key study variables*									
1. Parenting stress	79.27	19.45	—						
2. Gender^a^	0.54	0.50	−0.01	—					
3. Division of childcare labor discrepancy	0.77	0.63	0.19*	−0.20*	—				
4. DAS‐consensus	48.27	6.64	−0.23**	0.07	−0.34**	—			
5. DAS‐satisfaction	38.40	6.55	−0.19*	−0.19*	−0.19*	0.57**	—		
6. DAS‐cohesion	16.39	3.46	−0.24**	−0.25**	−0.25**	0.42**	0.40**	—	
7. DAS‐affectional expression	8.00	1.79	−0.02	−0.06	−0.06	0.47**	0.41**	0.35**	—
*Key covariates*									
Number of children	1.54	0.90	0.05	−0.01	−0.03	−0.03	0.04	−0.03	−0.11
Eldest child age	5.32	3.68	0.09	0.14*	0.14	−0.10	−0.18*	−0.04	−0.11
Paid working hours	29.59	18.41	−0.17*	0.18**	−0.17*	0.01	−0.06	−0.02	−0.04

*Note:* SD = standard deviation. *N* ranges from 156 to 226 given missingness. *N* represents the number of participants for each pair of zero‐order correlations. Higher scores of parenting stress indicate greater parenting stress. Higher scores of the division of childcare labor discrepancy indicate greater dissatisfaction of the current allocation of childcare tasks. Higher scores of DAS measures indicate greater relationship well‐being.

Abbreviation: DAS = dyadic adjustment scale.

^a^ 1 = transgender man and woman, 0 = nonbinary.

* *p* < 0.05.

** *p* < 0.01 (two‐tailed).

In terms of direct effect, greater parenting stress was associated with lower levels of couple relationship quality (*b* = −0.062, SE = 0.022, *p* = 0.005, *β* = −0.222), so H1 was supported. For the indirect effect, bootstrapping analyses showed that higher levels of parenting stress were associated with poorer relationship quality indirectly via giving rise to division of childcare labor discrepancy (*b* = −0.026, SE = 0.017, 95% CI [−0.074, −0.002], *β* = −0.095), which aligned with H2. The effect size of the indirect effect was about medium (Kenny 2017). The estimate of the *post hoc* power for the indirect effect was 0.994 (Kenny 2017), indicating adequacy in power.

**FIGURE 3:**
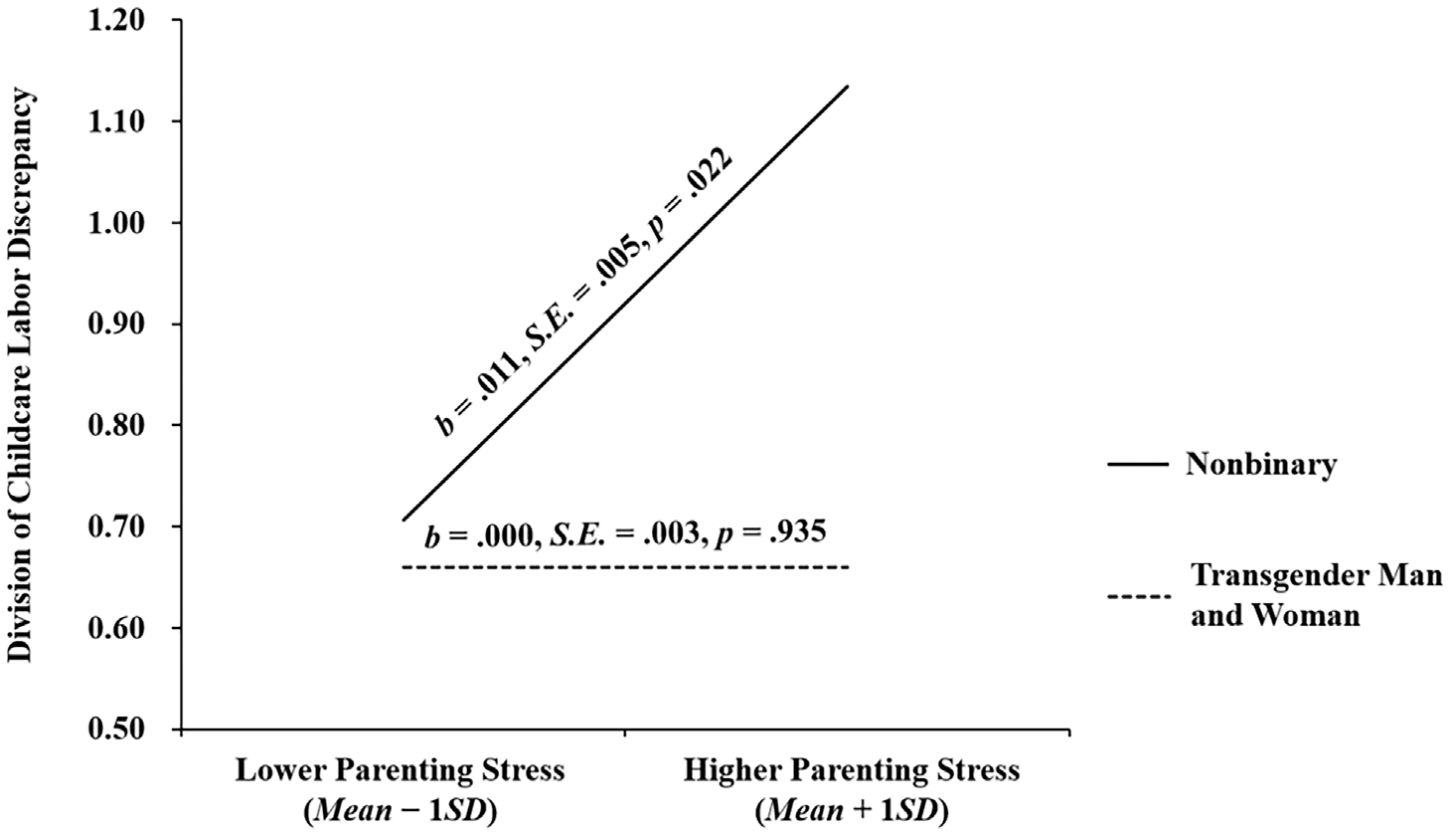
Probing simple slopes for the moderating role of gender identity.

Further, the interaction between parenting stress and gender identity significantly predicted the division of childcare labor discrepancy (*b* = −0.011, SE = 0.005, *p* = 0.040, *β* = −0.256). Simple slope analyses (see Figure 3) indicated that parenting stress was positively linked to the division of childcare labor discrepancy among nonbinary parents (*b* = 0.011, SE = 0.005, *p* = 0.022), but was not associated with the division of childcare labor discrepancy among transgender man and woman parents (*b* = 0.000, SE = 0.003, *p* = 0.935). Thus, H3 was supported.

For nonbinary parents, parenting stress was negatively related to couple relationship quality via a positive association with the division of childcare labor discrepancy (*b* = −0.026, SE = 0.017, 95% CI [−0.074, −0.002]). In contrast, for transgender man and woman parents, parenting stress was not related to relationship quality via the discrepancy (*b* = 0.001, SE = 0.008, 95% CI [−0.014, 0.017]). Thus, a conditional indirect pathway was identified as hypothesized: More experiences of parenting stress were associated with lower relationship quality through higher division of childcare labor discrepancy, but only among nonbinary parents.

## 4 Discussion

Grounded in the ecological model of coparenting (Feinberg 2003) and vulnerability‐stress‐adaptation model (Karney and Bradbury 1995), and informed by the concept of decentering heteronormativity (Oswald et al. 2005), this study investigated the mediating role of discrepancy in the division of childcare labor in the association between parenting stress and relationship quality, along with the moderating role of gender identity in the links between parenting stress and division of childcare labor discrepancy among TNB parents from Westernized countries. Most importantly, the results of our moderated mediation model identified a conditional indirect pathway. That is, greater exposure to parenting stress was associated with lower relationship quality through higher discrepancies in the division of childcare labor only among nonbinary TNB parents. This study expands upon previous research concerning TNB family processes through two pivotal contributions: by elucidating the underlying mechanism that may explain the negative association between parenting stress and relationship quality, and by examining more nuanced gender variations within the TNB group related to such dynamics.

Consistent with H1, the findings indicated that TNB parents with greater levels of parenting stress reported lower relationship quality. This result provided support for the spillover process from the parent–child domain to the couple domain (Cox et al. 2001; Cox and Paley 1997) in the context of gender‐diverse parent families. This finding is aligned with the existing evidence drawn from cisgender heterosexual samples (Berryhill et al. 2016; Chester and Blandon 2016; Ding et al. 2022; Durtschi et al. 2017; Hartley et al. 2018).

Our finding of identifying the discrepancy in the division of childcare labor as a destructive mechanism underlying the association between parenting stress and TNB parents' couple relationship quality supports H2. This result is consistent with the ecological model of coparenting, which highlights coparenting processes as key pathways through which parental adjustment influences interparental relationship functioning (Feinberg 2003). It is also aligned with empirical evidence. In a study using dyadic data from Korean parents of children with atopic dermatitis, the results demonstrated a mediation effect that fathers' parenting stress increased their perceived marital conflicts through undermining coparenting relationships with their partners (Han and Lee 2020). As for the specific aspect of the division of childcare labor in coparenting, extant research suggests that perceived fairness of the division of labor, a similar concept to the division of labor discrepancy (Cowan and Cowan 1988), functions as a pivotal intermediary factor connecting family processes and marital relationships (Lavee and Katz 2002; Mikula et al. 2012). For example, Newkirk et al. (2017) investigated 108 working‐class, dual‐earner heterosexual couples in the USA during their transition to parenthood and found that mothers' perceived fairness about division of childcare tasks mediated the association between perceived changes in childcare responsibilities upon returning to work and later relationship conflicts.

Consistent with H3, we also found the linkage between parenting stress and discrepancy in the division of childcare labor was shaped by gender identity, with increased parenting stress only predicting a greater discrepancy in the division of childcare labor among nonbinary parents rather than transgender man and woman parents. Building upon existing research concerning gender roles and satisfaction with the division of labor primarily within heterosexual couples (Kuo et al. 2017; Lavee and Katz 2002), our study applies the model of decentering heteronormativity by going beyond a binary perspective of conventional gender differences in domestic labor division. The current findings subvert ingrained perceptions of gender within the family and provide a more nuanced understanding of how gender identity in the TNB community interacts with experiences of parenting stress to have implications on their discrepancies in the division of childcare labor (Oswald et al. 2005). Previous literature demonstrated that nonbinary individuals were more inclined to embrace a fluid sense of gender compared to binary individuals (Bradford and Catalpa 2019). In the specific domain of parenting, research has indicated that children with nonbinary parents participate in a greater array of gender‐expansive activities compared to children with transgender man and woman parents (Riskind and Tornello 2022). This evidence suggests that nonbinary parents face increased pressure to challenge the hegemonic gender norms reflected in the childcare labor division (Pollitt et al. 2018), so they may be more intentional about distributing childcare responsibilities according to specific circumstances as appropriate. As a result, in our study, increased parenting stress was found to be associated with greater undesirable perceptions of childcare labor division among nonbinary parents, whereas transgender man and woman parents' perceived discrepancies in the division of childcare labor exhibited minimal correlation with parenting stress.

Most importantly, a conditional indirect pathway was identified such that only for nonbinary parents, reports of parenting stress were negatively associated with couple relationship quality through a positive association with division of childcare labor discrepancy. This pathway as a whole highlights the understudied intricacies of TNB parents' intersectional experiences of gender identity, intimate relationships, and parenthood. Such complexity is underscored by various well‐documented dynamics, including stress originating in the parent–child subsystem seeping into the couple relationship subsystem (Berryhill et al. 2016), discrepancies in the division of childcare labor as one intermediary component of coparenting dynamics (Feinberg 2003; Lavee and Katz 2002), and individual characteristics interacting with family stress to shape familial processes (Sun et al. 2017).

### 4.1 Theoretical Implications

Our findings have implications for both contemporary and classical theories of gendered labor. Integrating the ecological model of coparenting with the Vulnerability‐Stress‐Adaptation (VSA) model clarifies how extra‐dyadic stressors originating in the parenting domain can proliferate into the couple subsystem and shape childcare division expectations via prevailing gender norms, creating intra‐dyadic stressors that may be particularly salient for nonbinary parents. Consistent with calls for decentering heteronormativity within family scholarship (Oswald et al. 2005), the results suggest that expectations to adopt gendered parenting roles may be affirming for some transgender man and woman parents but experienced as non‐affirming by many nonbinary parents.

Classic accounts of gendered labor, such as Sex Role theory (Eagly 1987), further illuminate these dynamics: when gender is highly salient, such as in division of childcare labor, gender stereotypes and expectations shape caregivers' behavior and task allocation. Combined with our findings, this framework points to a socialization process whereby some transgender man and woman parents may adopt gendered parenting roles potentially to affirm identity, whereas nonbinary parents may be less likely or able to conform to those roles, producing different patterns of parenting stress and relationship outcomes.

### 4.2 Practical Implications

Our findings have important implications for family‐based interventions aimed at enhancing the relational well‐being of TNB parents. While core elements of existing coparenting programs (e.g., family foundations; Feinberg et al. 2010)—such as improving parental emotion regulation, couple communication, and conflict resolution—are broadly relevant, they may not be fully culturally responsive to gender‐diverse coparents. The presence of gender diversity can challenge hegemonic gender norms embedded in family processes, and intervention content should explicitly address those dynamics.

As our results suggest, the division of domestic labor emerges as a pronounced intervening point to promote couple relational outcomes for TNB parents. Hence, intervention components, such as structured exercises to negotiate a fair division of childcare and household tasks and training in constructive communication about labor sharing, may assist TNB parents in managing childrearing burdens and protecting couple well‐being. Moreover, the gender differences observed in this study highlight heterogeneity in gender ideology among TNB parents and suggest the need to identify subgroups who may benefit the most from targeted supports. Developing and testing affirming, culturally responsive intervention models that explicitly incorporate variations in gender identity and ideology represents a promising direction for future research and practice.

### 4.3 Limitations and Future Directions

Some limitations and future directions of this study should be noted. First, because mediation involves causal processes that unfold over time (Maxwell & Cole, 2007), the cross‐sectional and correlational nature of this study may bias estimates of both direct and indirect effects. Accordingly, we interpret our findings with caution and do not claim evidence of a causal process. Future research should employ longitudinal designs with time‐varying variables assessed at three or more time points and utilize advanced modeling approaches better suited to capturing longitudinal mediation processes, such as cross‐lagged panel, latent growth curve, or latent difference score models (O'Laughlin et al. 2018). Second, most participants self‐identified as white due to our sampling approach, suggesting that we need to be cautious about the generalizability of our results. Future research could consider oversampling underrepresented populations, such as people of color. Third, although the sample size was adequately powered to examine differences in the indirect effects of parenting stress on relationship quality between transgender man and woman and nonbinary parents, it was insufficient to analyze more nuanced gender identity categories within each group (e.g., transgender man, transgender woman, and agender) separately. Future research with larger and more diverse samples of TNB parents is needed to more comprehensively investigate how parenting stress, division of labor, couple relationship well‐being, and the associations among these variables may differ across the full spectrum of gender identities. Fourth, although prior studies have used the same measures of parenting stress (Gamarel et al. 2014), division of childcare labor (Tornello 2020), and couple relationship quality (Lampis et al. 2023) with TNB participants and have reported adequate psychometric properties, to the best of our knowledge, these measurements have not been systematically validated for use specifically with TNB populations. The lack of formal validation may constrain the interpretability and generalizability of our findings for TNB individuals. Future research is therefore needed to culturally adapt existing measures and, when necessary, develop new instruments that more fully capture the experiences and constructs relevant to TNB parents.

## 5 Conclusions

As our results suggest, parenting stress predicted relationship well‐being via division of childcare labor discrepancy, but only among nonbinary TNB parents. To the best of our knowledge, this study is the first to identify the division of childcare labor discrepancy as a destructive mechanism accounting for the negative association between parenting stress and TNB parents' couple relationship quality. This conditional indirect pathway alludes to an important finding within family science concerning TNB family processes, as it suggests that a more refined understanding is needed when exploring the interactions of gender identity, parenthood, and relationships within families headed by TNB parents. We see from our findings that nonbinary TNB parents may have a unique experience of increased pressure to push against heteronormative parental roles within the childcare labor division compared to binary TNB parents and may be more concerned about the distribution of childcare responsibilities. These complex, identity‐linked dynamics merit further empirical attention and should be considered in both future research and the development of culturally responsive, family‐centered interventions for TNB parents.

## Funding

The authors acknowledge financial support from The Pennsylvania State University‐Altoona Research Development Grant and The National Institute on Drug Abuse (T32 DA017629).

## Conflicts of Interest

The authors declare no conflicts of interest.

## Supporting information


**Tables S1–S2:** famp70141‐sup‐0001‐TableS1‐S2.docx.
