# Supplementary material for: Parenting Stress and Couple Relationship Quality Among Transgender and Nonbinary Parents: The Roles of Discrepancy in Division of Childcare Labor and Gender Identity
Source: Fam Process. 2026 Mar 26;65(2):e70141. doi: 10.1111/famp.70141 (PMC13021297; doi:10.1111/famp.70141)
Supplement: Supplementary file 1 — Tables S1–S2: famp70141‐sup‐0001‐TableS1‐S2.docx. [file FAMP-65-0-s001.docx]

**Supplemental Material**

**Table S1**

*Demographic Characteristics of Transgender and Nonbinary Parents and their Eldest Child (N = 228)*

| **Variables** | **Parent** | **Child** |
| --- | --- | --- |
|  | ***M* (*SD*) or *n* (%)** | ***M* (*SD*) or *n* (%)** |
| Age | 35.30 (6.67) | 5.32 (3.68) |
| Household income per year (in thousands) | 79.00 (70.77) |  |
| Paid working hours per week | 29.59 (18.41) |  |
| Length of relationships (in years) | 10.04 (5.47) |  |
| Number of children | 1.54 (.90) |  |
| Sex assigned at birth (female %) | 161 (70.93) | 96 (43.84) |
| Gender (%) |  |  |
| *Transgender woman* | 46 (20.18) |  |
| *Transgender man* | 48 (21.05) |  |
| *Woman* | 18 (7.89) |  |
| *Man* | 10 (4.39) |  |
| *Genderqueer* | 31 (13.60) |  |
| *Non-binary* | 26 (11.40) |  |
| *Gender nonconforming* | 12 (5.26) |  |
| *Agender* | 10 (4.39) |  |
| *Gender fluid* | 8 (3.51) |  |
| *Choose not to label* | 1 (.44) |  |
| *Additional gender identities* ^a^ | 14 (6.14) |  |
| Sexual orientation (%) |  |  |
| *Queer* | 57 (25.00) |  |
| *Pansexual* | 40 (17.54) |  |
| *Bisexual* | 38 (16.67) |  |
| *Lesbian* | 34 (14.91) |  |
| *Heterosexual* | 17 (7.46) |  |
| *Choose not to label* | 9 (3.95) |  |
| *Gay* | 8 (3.51) |  |
| *Asexual* | 7 (3.07) |  |
| *Demisexual* | 5 (2.19) |  |
| *Questioning* | 5 (2.19) |  |
| *Additional sexual orientation ^b^* | 8 (3.51) |  |
| Race/ethnicity (%) |  |  |
| *White/Caucasian* | 205 (89.91) | 178 (81.28) |
| *Biracial/Multiracial* | 7 (3.07) | 26 (11.87) |
| *Hispanic/Latino(a)* | 4 (1.75) | 7 (3.20) |
| *Filipino* | 2 (.88) | 2(.91) |
| *Additional races/ethnicities ^c^* | 10 (.4.39) | 6 (2.74) |
| **Variables** | **Parent** | **Child** |
|  | ***M* (*SD*) or *n* (%)** | ***M* (*SD*) or *n* (%)** |
|  |  |  |
| Relationship status (%) |  |  |
| *Married legally* | 152 (66.67) |  |
| *Committed relationship* | 32 (14.04) |  |
| *Engaged* | 14 (6.14) |  |
| *Polyamorous* | 7 (3.07) |  |
| *Married but no legal recognition* | 6(2.63) |  |
| *Dating* | 5 (2.19) |  |
| *Additional relationship status ^d^* | 12 (5.26) |  |
| Education (%) |  |  |
| *Less than high school* | 2 (.89) |  |
| *High school/GED* | 14(6.14) |  |
| *Vocational/Trade school* | 5 (2.19) |  |
| *Associates degree/2 years* | 23 (10.09) |  |
| *Bachelor’s degree/4 years* | 106 (47.37) |  |
| *Graduate degree* | 76 (33.33) |  |
| Country of residence |  |  |
| *United States* | 168 (73.68) |  |
| *Canada* | 22 (9.65) |  |
| *United Kingdom* | 14 (6.14) |  |
| *Australia* | 12 (5.26) |  |
| *Additional countries of residence ^e^* | 12 (5.26) |  |

*Note.* Not all numbers will total to 100% due to rounding. The parent’s sex assigned at birth, the eldest child’s sex assigned at birth, and the eldest child’s race/ethnicity have missing values, so a valid percentage was used for the above characteristics.

^a^ Additional gender identities include androgyne, transfeminine, transmasculine, etc.

^b^ Additional sexual orientation includes androsexual, queer demisexual, etc.

^c^ Additional race/ethnicities include American Indian/Alaskan Native, Black/African American, etc.

^d^ Additional relationship status includes civil partnership, in the process of divorce, etc.

^e^ Additional countries of residence include Germany, Finland, Sweden, Norway, and Switzerland.

**Table S2**

*Model Comparison Steps for Multigroup SEM Analysis Comparing Transgender Man and Woman and Nonbinary Parents*

| **Step** | **Model Description** | **χ²** | **df** | **Δχ²** | **Δdf** | ***p*-value** | **Model Decision** |
| --- | --- | --- | --- | --- | --- | --- | --- |
| 1 | Unconstrained model | 30.52 | 22 | − | − | − | − |
| 2 | Constrain path from X to M | 35.34 | 23 | 4.82 | 1 | **0.03** | This constraint worsens model fit → free the path from X to M |
| 3 | Constrain path from M to Y | 30.70 | 23 | 0.18 | 1 | 0.68 | This constraint does not worsen model fit → constrain the path from X to M |
| 4 | Constrain path from M to Y + path from X to Y | 30.83 | 24 | 0.13 | 1 | 0.71 | This constraint does not worsen model fit → constrain the path from X to M and from X to Y |

*Note.* X = parenting stress, M = division of childcare labor discrepancy, Y = relationship quality. Δχ² and Δdf represent the change in χ² and degrees of freedom compared to the preceding model. Significant *p*-values are in bold and indicate that constraining the path significantly worsens model fit.
